# Geospatial Access to CAR-T Clinical Trials for Non-Hodgkin Lymphoma for Persons With HIV

**DOI:** 10.1001/jamanetworkopen.2026.14265

**Published:** 2026-05-22

**Authors:** Luke Maillie, Matthew Sisk, Anna E. Coghill, Nathan Van Bibber, Carmen E. Guerra, David M. Aboulafia, Thomas M. Atkinson, Victor Velazquez, Stefan K. Barta

**Affiliations:** 1 Department of Medicine, University of Pennsylvania, Philadelphia; 2 Abramson Cancer Center, University of Pennsylvania, Philadelphia; 3 Lucy Family Institute for Data & Society, University of Notre Dame, Notre Dame, Indiana; 4 H. Lee Moffitt Cancer Center and Research Institute, Tampa, Florida; 5 Virginia Mason Medical Center, Seattle, Washington; 6 Community Advisory Board, University of Washington Positive Research, Seattle; 7 Memorial Sloan Kettering Cancer Center, New York, New York

## Abstract

**Importance:**

Chimeric antigen receptor T-cell (CAR-T) therapy is a paradigm-changing therapy in treating non-Hodgkin lymphoma (NHL). Although NHL is a leading cause of cancer-attributable deaths for persons living with HIV (PWH) in high-income countries, PWH are frequently excluded from CAR-T clinical trials.

**Objective:**

To explore access to CAR-T trials for the general population and PWH.

**Design, Setting, and Participants:**

This cross-sectional study queried ClinicalTrials.gov for all interventional CAR-T clinical trials for the treatment of NHL. Zip codes for each trial site were extracted, and travel time to the nearest location was calculated. Trials included had at least 1 trial site in the contiguous US, and were actively recruiting trials for adult patients (aged ≥18 years) as of May 20, 2025.

**Exposure:**

All trials were reviewed for relevance and eligibility of PWH.

**Main Outcomes and Measures:**

The primary outcomes were median population-weighted travel time to the nearest trial and 1-hour and 3-hour access for the general population, HIV-inclusive trials, and HIV-exclusive trials. Proportions were compared using a χ^2^ test, and continuous median travel times were compared using unpaired *t* tests.

**Results:**

In total, 254 trials were eligible for review, and 80 met criteria for inclusion with 11 (13.8%) trials including PWH, 58 (72.5%) excluding PWH, and 11 (13.8%) not mentioning HIV. The median (IQR) population-weighted travel time was 0.73 (0.36-1.64) hours for the general population, 1.15 (0.49-2.38) hours for trials including PWH, and 0.84 (0.40-1.91) hours for trials excluding PWH. Compared with trials that excluded PWH, trials that included PWH had significantly lower 1-hour (46.07% [95% CI, 46.06%-46.07%] vs 55.27% [95% CI, 55.27%-55.28%]; *P* < .001) and 3-hour (82.22% [95% CI, 82.22%-82.23%] vs 87.76% [95% CI, 87.75%-87.76%]; *P* < .001) access. Travel time in the South was significantly longer for trials that included PWH compared with trials that excluded PWH (median [IQR], 1.70 [0.69-2.99] hours vs 0.92 [0.46-2.00] hours; *P* < .001).

**Conclusions and Relevance:**

This cross-sectional study of travel time to CAR-T trials found that PWH had to travel significantly longer than the general population to reach the nearest trial. Despite efforts to reduce clinical trial exclusion based solely on HIV status, access for PWH remains disproportionately lower compared with the general population, and particularly poor in the South, where there is the highest prevalence of HIV. Further efforts to increase access to trials for underserved and underrepresented populations, particularly for PWH, are needed.

## Key Points

**Question:**

Do persons living with HIV (PWH) have the same access to chimeric antigen receptor T-cell (CAR-T) clinical trials for non-Hodgkin lymphoma (NHL) as persons without HIV?

**Findings:**

In this cross-sectional study of 80 clinical trials, PWH had to travel significantly longer to the nearest CAR-T clinical trial for NHL (1.15 vs 0.84 hours). The greatest regional difference was in the South, where PWH had to travel 1.70 hours compared with 0.92 hours for persons without HIV.

**Meaning:**

These findings suggest that further efforts are needed to expand access to CAR-T clinical trials for NHL for PWH, particularly in the South.

## Teaser

This cross-sectional study investigates whether persons living with HIV have the same access to chimeric antigen receptor T-cell (CAR-T) clinical trial therapy for non-Hodgkin lymphoma as persons without HIV.

## Introduction

Chimeric antigen receptor T-cell (CAR-T) therapy is a treatment that utilizes engineered T-cells to target antigens on tumor cells and has become a paradigm-changing intervention for patients with relapsed or refractory non-Hodgkin lymphoma (NHL).^1^ Three pivotal clinical trials^2,3,4^ led to the US Food and Drug Administration (FDA) approval of CAR-T as third-line therapies for relapsed or refractory NHL. Follow-up trials^5,6,7,8,9^ supported approval of CAR-T for second-line treatment of relapsed or refractory NHL.

Prior studies have shown substantial regional disparities in travel time to cancer clinical trials,^10^ and increased travel time to trials results in significantly lower odds of trial participation.^11^ Major geospatial disparities in access, specifically for CAR-T clinical trials, have already been demonstrated for patients with multiple myeloma,^12^ with 1 study linking increased travel distance with increased mortality.^13^ Another study examining access to standard of care CAR-T for relapsed or refractory large B-cell lymphoma using Medicare data shows that 85% of patients who received CAR-T lived within 2 hours of their treatment center, and patients living between 2 to 4 hours away were 40% less likely to receive CAR-T.^14^

Access to cutting-edge clinical trials for NHL is of particular importance for persons living with HIV (PWH). As the proportion of AIDS-related mortality in the US and Europe continues to fall over time, declining from 49% over the 1996 to 1999 period to 16% over the 2016 to 2020 period, the proportion of mortality attributable to cancer has risen from 5% to 19%.^15^ Furthermore, cancer mortality rates are higher for PWH compared with persons without HIV,^16,17,18^ and NHL is a leading cause of cancer-attributable deaths among PWH in the US, particularly among younger adults.^17^ The reasons for the increased rates of mortality are likely multifactorial, including both increased incidence of some malignant cancers in PWH^17^ and lower levels of access to cancer care compared with persons without HIV.^19,20^

Despite this, PWH have historically been excluded from clinical trials for cancer therapeutics.^21^ Of note, overall survival for PWH and NHL has improved over time and is reported to approach that for people without HIV for patients enrolled in certain cancer clinical trials, emphasizing the importance of their inclusion.^22^ A recent study demonstrated that PWH with NHL are eligible for trials for CAR-T at significantly lower rates than persons without HIV with 62% of all CAR-T trials excluding PWH.^23^ Although longer travel times are associated with lower rates of access and worse outcomes for CAR-T, limited information exists examining travel time to CAR-T clinical trials for NHL specific to PWH within the US. This study examines whether PWH have equal access to CAR-T clinical trials for NHL as compared with the general population.

## Methods

In this cross-sectional study, we surveyed the National Institute of Health’s clinical trials database, ClinicalTrials.gov, for all trials related to the treatment of NHL with CAR-T therapies. Searches for *lymphoma* and *non-Hodgkin lymphoma* plus *CAR-T*, *chimeric-antigen receptor therapy*, and *CAR-T therapy* were used. Trials were then individually reviewed for eligibility for inclusion and relevance. All CAR-T interventional trials focused on adult patients (aged ≥18 years) with at least 1 trial site in the US were included for review. Only trials that were actively seeking participants on May 20, 2025, defined as either not yet recruiting or recruiting as per ClinicalTrials.gov guidelines, were included. This work was exempt by the institutional review board at the University of Pennsylvania and followed Strengthening the Reporting of Observational Studies in Epidemiology (STROBE) reporting guideline for cross-sectional studies.

### Defining HIV Inclusion

Eligibility criteria for each trial were examined to see whether PWH were included or excluded. If a trial did not mention HIV, we searched for a study publication or a published study protocol to see if further inclusion and exclusion criteria mentioning HIV were available. Any trial that mentioned a diagnosis of HIV as an exclusion criterion was considered to exclude PWH. Trials that mentioned secondary immunodeficiency, chronic viral infections, or active infection without additional specification were also considered to exclude PWH. If we were unable to locate information on HIV eligibility, we conservatively assumed that the trial allowed inclusion of PWH to ensure that maximal possible access was measured. A sensitivity analysis examining access for studies that explicitly listed HIV as an inclusion criterion was also performed.

### Defining Travel Time

We extracted zip codes of each trial site for every trial. A transportation network using publicly available roadway network data that included road speeds was generated using Open Source Routine Machine.^24^ All further analysis was conducted in R version 2023.12 (R Project for Statistical Computing). We then used this transportation network to find the nearest zip code tabulation area (ZCTA) centroid or geocoded zip code to all actively enrolling CAR-T clinical trial sites for every ZCTA in the US and calculate the travel time between each ZCTA and the nearest trial site. We used geometric centroids of ZCTAs as reference points for all travel time calculations, and adult population data from the 2020 US Census were used to calculate population-weighted travel time.^36^

We also extracted demographic data from the 2016 to 2020 American Community Survey.^36^ This included race and ethnicity (American Indian or Alaska Native, Asian, Black, Hispanic, and White), socioeconomic status defined by median household income divided into quartiles, and insurance status (Medicare only, Medicaid only, private insurance only, and uninsured). In the American Community Survey, participants self-define their race and ethnicity. Race and ethnicity were included in this study to assess for disparities across these categories. Only insurance plans defined as exclusively private vs public (Medicare and Medicaid) were considered for comparison. Since insurance coverage rates were not available for the adult population, rates for the general population for each zip code were applied to the adult population to estimate coverage. Regions (Northeast, Midwest, South, and West) were defined according to US Census definitions. Only trial sites in the contiguous US including the District of Colombia were used for analysis.

### Statistical Analysis

Proportions were compared using a χ^2^ test, and continuous median travel times were compared using unpaired *t* tests. Statistical significance was defined as 2-sided *P* < .05. Specifically, the shortest travel time between every ZCTA in the US and the nearest CAR-T clinical trial was compared according to HIV inclusion and exclusion status. Travel time to a CAR-T clinical trial was also analyzed as a categorical variable, with comparisons by study group conducted using 1-hour and 3-hour access categories, consistent with prior access definitions.^25,26^ Three hours was selected to represent the maximum reasonable amount of time a patient can travel to a clinical trial site and still return home in a single day.^26^ Statistical analysis was completed using R.

Three separate study groups were compared: all CAR-T clinical trials (general population), trials that included PWH (HIV included), and trials that excluded PWH (HIV excluded). All trials included travel time to nearest trial for all eligible trials, regardless of whether PWH were eligible. HIV included measured access to the nearest trials that included PWH, and HIV excluded measured access to the nearest trials that excluded PWH but were open to the general population. Prevalence of HIV from 2020 AIDSVu data were used to calculate prevalence of HIV included trials per 100 000 HIV population.^27^

## Results

A total of 254 CAR-T clinical trials were eligible for review, with 159 (62.6%) of those studies meeting criteria for inclusion, with 80 (50.3%) actively recruiting (eFigure 1 in Supplement 1). In total, 22 studies (27.5%) were considered to include PWH, which included 11 trials (13.8%) that specifically mentioned HIV as an inclusion criterion and 11 (13.8%) with no mention of HIV status, and 58 studies (72.5%) were classified as specifically excluding PWH. Of the 58 trials excluding PWH, 1 (1.7%) mentioned a reason for excluding PWH (concern that immunosuppression from therapy with concurrent HIV posed an unacceptable risk) and 57 (98.3%) did not mention a reason for exclusion. Seventy trials (87.5%) used in-house CAR-T and 10 (12.5%) used commercially available CAR-T; 61 (76.3%) used CAR-T alone and 19 (23.8%) used CAR-T with at least 1 other therapy.

A breakdown of trial phase and funder by HIV inclusion criteria is shown in Table 1. A significantly increased proportion of phase 2 trials compared with phase 1 trials excluded PWH (17 trials [65.4%] vs 6 trials [9.4%]). We observed no significant difference in the inclusion of PWH by trial funder-type.

**Table 1.** Characteristics of CAR-T Clinical Trials That Included or Excluded PWH

Characteristic	Trials, No. (%) (N = 80)		Total, No.
	Included PWH	Excluded PWH	
Phase			
Early phase 1	1 (50.0)	1 (50.0)	2
Phase 1	58 (90.6)	6 (9.4)	64
Phase 2	9 (34.6)	17 (65.4)	26
Phase 3	0	1 (100.0)	1
Funder			
National Institutes of Health	6 (42.9)	8 (57.1)	14
Industry	9 (25.7)	26 (74.3)	35
Others^a^	17 (29.3)	43 (74.1)	58

Abbreviations: CAR-T, chimeric antigen receptor T-cell; PWH, persons with HIV.

^a^ Others refers to individuals, universities, and community-based organizations. Total number exceeds 80 since several trials had multiple phases and multiple funders.

Among the 80 actively recruiting CAR-T clinical trials, we identified 362 US trial sites, 125 (34.5%) including PWH and 237 (65.5%) excluding PWH. There were 175 unique zip codes represented among the 362 total clinical trial sites. The median number of sites per trial was 1 (range, 1-76). Forty-four trials (55.0%) took place at only 1 site. The median number of trials by state was 5 (range, 0-41). Ten states had 0 actively recruiting trial sites (eTable 1 in Supplement 1).

### HIV-Inclusive Trials 

**Figure 1.**
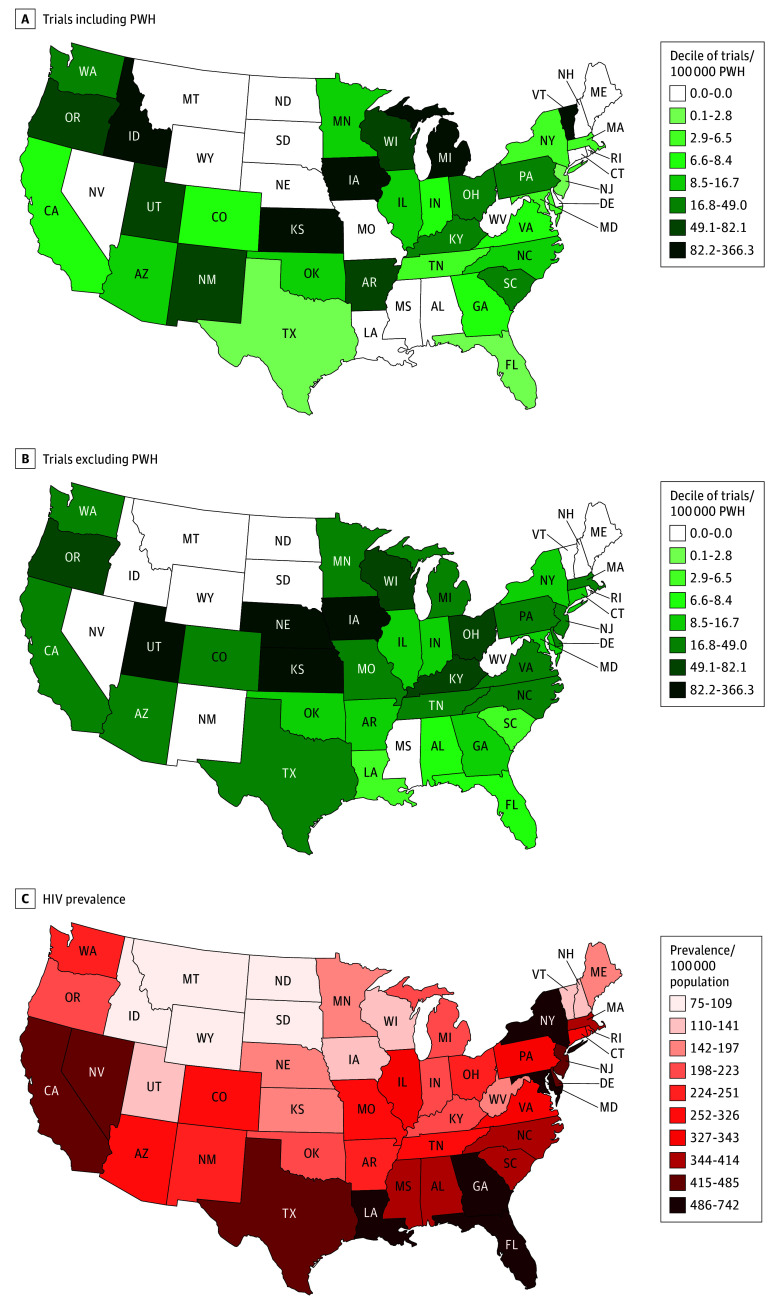
Maps of Prevalence of Trials by HIV Inclusion and/or Exclusion A and B, Color density in the key for each range is based on deciles of trials per 100 000 persons with HIV (PWH) calculated for trials that included PWH. The same deciles were applied to trials that excluded PWH to allow for direct comparison. The lower 3 deciles were consolidated into 1 group since they all had zero trials. Given the same denominator was used for both HIV-inclusive and HIV-exclusive trials, differences in density by HIV exclusion status for larger states with higher HIV prevalence indicate more non-HIV-inclusive trials. Additionally, the presence of any trial in states with smaller populations leads to an apparently higher density of trials. C, Prevalence of HIV by state per 100 000 population is shown for reference based on 2022 AIDSVu data.

For the 22 CAR-T clinical trials (125 trial sites) that were classified as inclusive of PWH , the median number of sites per trial was 1 (range, 1-76 sites) (Figure 1). There were 97 unique zip codes among the 125 trial sites. Fifty-five trial sites (44.0%) were located at a National Cancer Institute (NCI)–designated cancer center. The median number of HIV-inclusive CAR-T clinical trial sites by state was 1 (range, 0-17), with 17 states having 0 actively recruiting sites.

### HIV-Exclusive Trials 

For the 58 CAR-T clinical trials (237 trial sites) that excluded PWH, the median number of sites per trial was 1 (range, 1-46) (Figure 1). There were 118 unique zip codes among the 237 trial sites, with the maximum number of HIV-excluding CAR-T clinical trials in 1 zip code being 24. A total of 138 trial sites (58.2%) were located at an NCI-designated cancer center. This was a significantly higher proportion compared with trial sites that were HIV-inclusive (*P* = .01). The median number of HIV-excluding CAR-T clinical trial sites by state was 3 (range, 0-37), and 13 states had 0 actively recruiting sites.

### Access to CAR-T Clinical Trials by HIV Status

**Figure 2.**
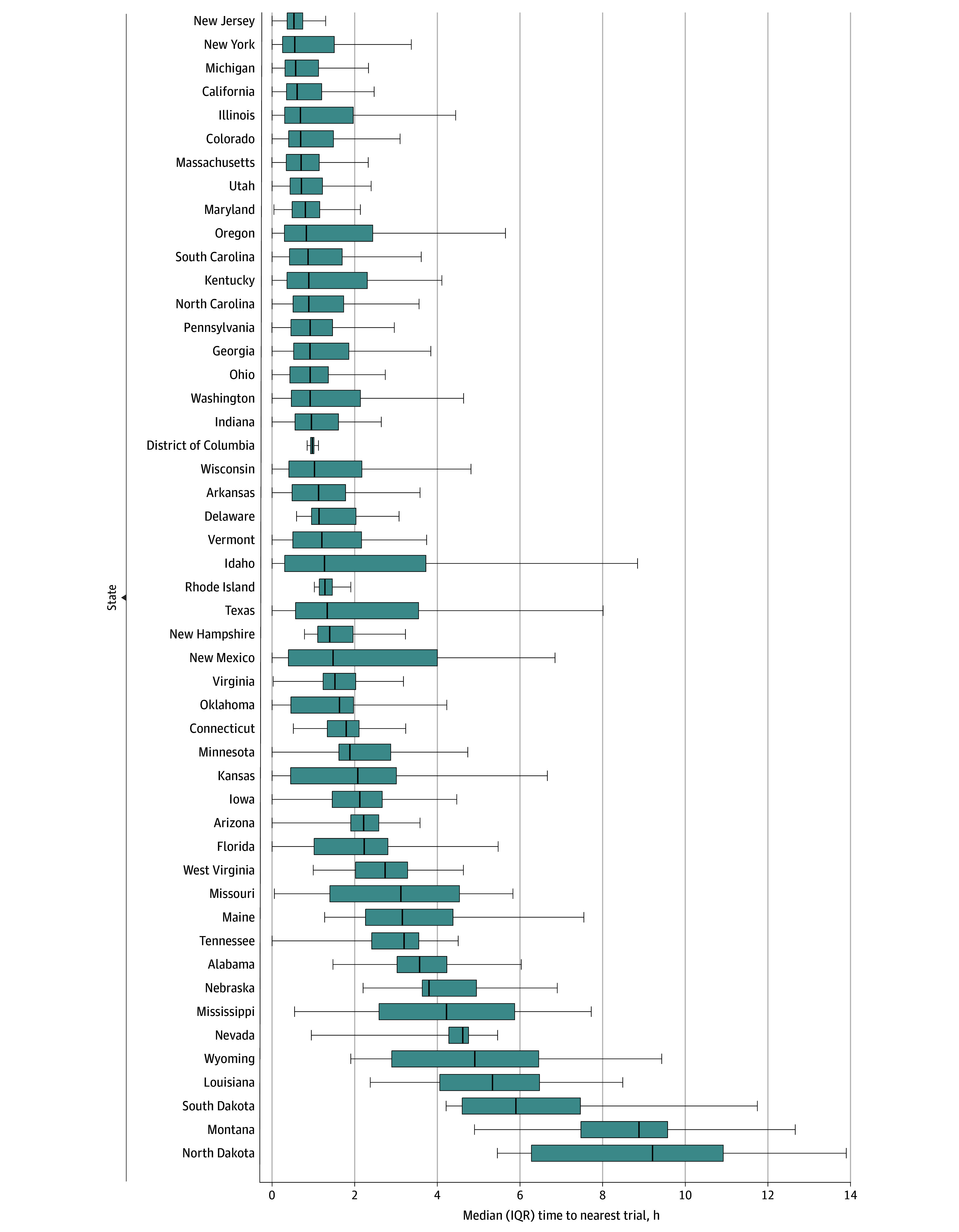
Box Plot of Variability by State of Time to Nearest Clinical Trial for Trials That Include PWH Figure depicts variability by state of median and IQR travel time to nearest clinical trial. Left whisker depicts the minimum, or if the value exceeded 1.5 times the IQR from the first quartile. 1.5 times the IQR was used instead. The left box edge depicts the first quartile. The center line is the median. The right box edge depicts the third quartile. The right whisker depicts the maximum or, if the value exceeded 1.5 times the IQR from the third quartile, 1.5 times the IQR was used instead. For the purpose of graphical representation only, outliers were censored and excluded in cases where the time exceeded 1.5 times the IQR. PWH indicates persons with HIV.

Median travel time by state was calculated for CAR-T clinical trials including PWH (Figure 2) and CAR-T clinical trials excluding PWH. We observed notable variability by state (eTables 2-4 in Supplement 1). Overall, the median (IQR) population-weighted travel time to the nearest CAR-T clinical trial was 0.73 (0.36-1.64) hours for 255.9 million adults in the contiguous US; 59.8% of the population (153.1 million people) were within 1 hour of the nearest actively enrolling CAR-T clinical trial, 81.1% (207.6 million) were within 2 hours, and 91.2% (233.3 million) were within 3 hours. Table 2 presents the results by demographic features.

**Table 2.** Travel Time to Nearest Clinical Trial for the General Population

Characteristic	Population, No. (millions) (%)	Population-weighted travel time, median (IQR), h^a^	Access, % (95% CI)	
			1 h	3 h
Total population	255.9 (100)	0.73 (0.36-1.64)	59.82 (59.82-59.83)	91.18 (91.17-91.18)
Region				
Northeast	45.8 (17.9)	0.55 (0.27-1.26)	66.99 (66.97-67.00)	97.42 (97.42-97.43)
Midwest	53.4 (20.9)	0.84 (0.39-1.72)	56.06 (56.05-56.07)	92.94 (92.93-92.94)
South	97.6 (38.1)	0.85 (0.44-1.81)	55.49 (55.48-55.50)	91.64 (91.63-91.64)
West	59.1 (23.1)	0.58 (0.33-1.64)	64.95 (64.93-64.96)	83.98 (83.97-83.99)
Race and ethnicity				
American Indian or Alaska Native	6.8 (2.7)	0.95 (0.40-2.06)	51.61 (51.57-51.64)	85.39 (85.36-85.42)
Asian	17.4 (6.8)	0.42 (0.26-0.70)	83.09 (83.07-83.11)	95.88 (95.87-95.89)
Black	33.9 (13.3)	0.52 (0.28-1.25)	69.92 (69.90-69.93)	94.88 (94.87-94.89)
Hispanic	43.1 (16.8)	0.52 (0.29-1.30)	69.18 (69.17-69.20)	88.95 (88.94-88.96)
White	185.0 (72.3)	0.86 (0.42-1.79)	54.89 (54.88-54.90)	90.32 (90.32-90.32)
Socioeconomic status, quartile^b^				
First (lowest; <$50 828)	NA	1.32 (0.37-2.33)	41.35 (41.34-41.36)	85.89 (85.88-85.90)
Second ($50 828-$64 678)	NA	1.04 (0.42-1.99)	48.88 (48.87-48.90)	88.26 (88.26-88.27)
Third ($64 679-$85 188)	NA	0.67 (0.37-1.38)	64.56 (64.55-64.58)	92.81 (92.80-92.81)
Fourth (highest; >$85 189)	NA	0.50 (0.31-0.77)	85.04 (85.04-85.05)	97.98 (97.97-97.98)
Health insurance				
Medicare only	14.1 (5.5)	0.85 (0.40-1.79)	55.27 (55.25-55.30)	90.43 (90.42-90.45)
Medicaid only	37.2 (14.5)	0.76 (0.33-1.76)	57.36 (57.34-57.37)	89.87 (89.86-89.88)
Private only	139.1 (54.4)	0.68 (0.35-1.49)	63.14 (63.13-63.15)	92.32 (92.32-92.32)
Uninsured	23.9 (9.3)	0.75 (0.35-1.81)	58.15 (58.13-58.17)	88.61 (88.60-88.62)

Abbreviation: NA, not applicable.

^a^ Data are the median population-weighted travel time to nearest clinical trial for the general population for actively enrolling clinical trials as well as 1-hour and 3-hour access. Results are shown for the entire population as well as by region, race and ethnicity, socioeconomic status, and health insurance coverage.

^b^ Socioeconomic status is based on quartiles of annual median household income for each zip code.

The median (IQR) population-weighted travel time for trials including PWH was 1.15 (0.49-2.38) hours, with trials including PWH having 1-hour access of 46.07% (95% CI, 46.06%-46.07%) of the population, representing 117.9 million US adults within 1 hour of the nearest trial, and 3-hour access of 82.22% (95% CI, 82.22%-82.23%), representing 210.4 million US adults within 3 hours of the nearest trial. Overall, 17.78% (95% CI, 17.77%-17.78%) of the population, representing 45.5 million US adults, lived beyond 3 hours from the nearest HIV-inclusive trial. The median (IQR) population-weighted travel time for trials excluding PWH was 0.84 (0.40-1.91) hours, which was statistically significantly shorter than for HIV-inclusive CAR-T clinical trials (*P* < .001), with 55.27% (95% CI, 55.27%-55.28%) of the general population, representing 141.4 million US adults, having 1-hour access to a trial and 87.76% (95% CI, 87.75%-87.76%) of the general population, representing 224.5 million US adults, having 3-hour access to a trial. Sensitivity analysis, in which only trials that explicitly mentioned including PWH were considered to included PWH, showed a median (IQR) population-weighted travel time of 1.59 (0.64-3.27) hours, with 36.86% (95% CI, 36.86%-36.87%) of the population, representing 94.3 million US adults, having 1-hour access and 71.87% (95% CI, 71.86%-71.88%) of the population, representing 183.4 million US adults, having 3-hour access.

Table 3 depicts these results by US region and demographic variables. Participants living in the South had to travel 2.2 times as long and the population in the Midwest had to travel 1.5 times as long to reach a trial that included PWH compared with the Northeast. Participants in the South also had to travel almost twice as far to reach a trial that included PWH compared with a trial that excluded PWH (median [IQR], 1.70 [0.69-2.99] hours vs 0.92 [0.46-2.00] hours). One-hour and 3-hour access to trials that included PWH compared with trials that excluded PWH was significantly worse for every race and ethnicity, socioeconomic status, and insurance type and for every region except for 3-hour access in Northeast (96.6% [44.2 million US adults] vs 94.3% [43.2 million US adults]) and West (83.0% [49.0 million US adults] vs 79.6% [47.9 million US adults]) although the absolute difference was minimal.

**Table 3.** Travel Time to Nearest Clinical Trial by Inclusion or Exclusion of PWH

Characteristic	Population-weighted travel time, median (IQR), h^a^		1-h Access, % (95% CI)			3-h Access, % (95% CI)		
	PWH included	PWH excluded	PWH included	PWH excluded	*P* value	PWH included	PWH excluded	*P* value
Total population	1.15 (0.49-2.38)	0.84 (0.40-1.91)	46.07 (46.06-46.07)	55.27 (55.27-55.28)	<.001	82.22 (82.22-82.23)	87.76 (87.75-87.76)	<.001
Region								
Northeast	0.77 (0.38-1.50)	0.57 (0.27-1.38)	58.46 (58.45-58.47)	64.65 (64.64-64.67)	<.001	96.55 (96.55-96.56)	94.31 (94.30-94.31)	<.001
Midwest	1.17 (0.48-2.28)	1.04 (0.46-2.05)	45.22 (45.21-45.23)	48.67 (48.65-48.68)	<.001	82.20 (82.19-82.21)	90.03 (90.02-90.04)	<.001
South	1.70 (0.69-2.99)	0.92 (0.46-2.00)	35.80 (35.80-35.81)	52.68 (52.67-52.69)	<.001	75.11 (75.10-75.12)	88.43 (88.42-88.43)	<.001
West	0.86 (0.40-2.20)	0.69 (0.37-2.13)	54.24 (54.23-54.25)	58.30 (58.29-58.32)	<.001	82.91 (82.90-82.92)	79.57 (79.56-79.58)	<.001
Race and ethnicity								
American Indian or Alaska Native	1.44 (0.55-2.78)	1.14 (0.45-2.55)	39.54 (39.51-39.58)	46.65 (46.61-46.68)	<.001	77.39 (77.36-77.42)	79.85 (79.82-79.88)	<.001
Asian	0.57 (0.33-1.26)	0.45 (0.28-0.81)	68.01 (67.99-68.04)	79.10 (79.08-79.12)	<.001	91.83 (91.82-91.84)	94.55 (94.54-95.56)	<.001
Black	0.82 (0.38-2.08)	0.57 (0.30-1.50)	55.36 (55.35-55.38)	66.19 (66.18-66.21)	<.001	85.02 (85.01-85.04)	92.62 (92.61-92.63)	<.001
Hispanic	0.84 (0.39-2.30)	0.57 (0.31-1.55)	54.44 (54.43-54.47)	65.53 (65.51-65.54)	<.001	81.80 (81.79-81.82)	86.59 (86.58-86.60)	<.001
White	1.32 (0.57-2.53)	1.00 (0.46-2.08)	41.26 (41.25-41.27)	50.03 (50.03-50.04)	<.001	80.67 (80.67-80.68)	86.43 (86.42-86.43)	<.001
Socioeconomic status, quartile^b^								
First (lowest; $50 828)	1.80 (0.55-3.06)	1.51 (0.44-2.67)	31.84 (31.83-31.86)	37.01 (37.00-37.02)	<.001	74.02 (74.00-74.03)	80.90 (80.89-80.91)	<.001
Second ($50 828-$64 678)	1.54 (0.58-2.82)	1.22 (0.48-2.35)	37.23 (37.22-37.24)	43.48 (43.47-43.49)	<.001	77.59 (77.58-77.60)	83.74 (83.73-83.75)	<.001
Third ($64 679-$85 188)	1.01 (0.47-2.14)	0.76 (0.40-1.64)	49.71 (49.70-49.72)	59.48 (59.47-59.49)	<.001	84.52 (84.51-84.53)	89.83 (89.83-89.84)	<.001
Fourth (highest; >$85 189)	0.73 (0.44-1.26)	0.53 (0.33-0.83)	65.96 (65.95-65.97)	81.65 (81.64-81.66)	<.001	92.98 (92.98-92.99)	96.87 (96.87-96.87)	<.001
Health insurance								
Medicare only	1.32 (0.53-2.58)	0.96 (0.43-2.05)	41.84 (41.82-41.87)	51.22 (51.20-51.25)	<.001	80.54 (80.52-80.56)	86.69 (86.67-86.71)	<.001
Medicaid only	1.16 (0.43-2.41)	0.89 (0.37-2.05)	45.88 (45.87-45.90)	52.72 (52.70-52.74)	<.001	81.67 (81.66-81.68)	86.32 (86.31-86.33)	<.001
Private only	1.06 (0.48-2.26)	0.76 (0.39-1.76)	48.51 (48.50-48.52)	58.51 (58.50-58.52)	<.001	83.66 (83.65-83.67)	89.17 (89.16-89.17)	<.001
Uninsured	1.25 (0.47-2.67)	0.84 (0.38-2.08)	44.38 (44.36-44.40)	54.30 (54.28-54.32)	<.001	78.49 (78.48-78.51)	85.13 (85.11-85.14)	<.001

Abbreviation: PWH, people with HIV.

^a^ Comparison of the median population-weighted travel time to nearest chimeric antigen receptor T-cell clinical trial between trials enrolling PWH and trials that excluded PWH, as well as 1-hour and 3-hour access metrics by region, race and ethnicity, socioeconomic status, and health insurance coverage.

^b^ Socioeconomic status is based on quartiles of median household income for each zip code.

There was also an inverse association of median household income and travel time to nearest trial site (ie, higher income was associated with shorter travel time) in both the HIV included and HIV excluded models, so further description of travel time by demographic and income variables was performed for each US region (eTable 5 in Supplement 1). This analysis demonstrated that this trend of lower income being associated with longer travel time held true for each region. A map depicting access to trials including PWH and trials excluding PWH is shown in eFigure 2 in Supplement 1.

## Discussion

To our knowledge, this cross-sectional study is the first to examine travel time to CAR-T clinical trials for NHL in the US by HIV status, despite the association between HIV and lymphoma and the documented lack of access to cutting-edge clinical trials among the HIV and cancer population. We observed that only 27.5% of CAR-T clinical trials actively enrolling at time of this study were inclusive of PWH, a clear gap in access that necessitates attention.

Overall, this study showed median travel time of 0.73 hours for CAR-T clinical trial access. Most CAR-T trials require participants to live within 1.5 hours of a trial center, and we found that over 1 in 4 people (27.6%) lived beyond this distance. PWH had a significantly longer median travel time compared with persons without HIV (1.15 hours vs 0.84 hours). Moreover, trials that included PWH had significantly worse 1-hour (46.07% vs 55.27%; *P* < .001) and 3-hour (82.22% vs 87.76%; *P* < .001) access compared with trials that excluded PWH. If we considered only trials that explicitly mentioned including PWH, median travel time increased to 1.59 hours and 1-hour and 3-hour access fell to 36.86% and 71.87%, respectively. Persons in the South, where HIV prevalence is highest, had to travel almost twice as long to reach a trial that included PWH compared with participants without HIV (1.70 hours vs 0.92 hours). In summary, nearly 1 in 5 adults—17.8% or 45.5 million people—in the US lives over 3 hours away from a CAR-T trial for NHL that includes PWH.

A study by Olivieri et al^23^ examined the inclusion of PWH in clinical trials for aggressive subtypes of NHL from 2014 to 2025 and specifically examined access to CAR-T trials. They found an inclusion rate of 38% for CAR-T trials, slightly higher than our total rate of 27.5%. This lower rate in our study is likely due to our study including all cases of NHL, a different study time period, and searching for study protocols outside of ClinicalTrials.gov.

We found that most HIV-inclusive CAR-T trials were phase 1 trials. Phase 2 trials excluded PWH at a significantly higher rate compared with phase 1 trials as well. The sole phase 3 trial excluded PWH as well. The reason for lower inclusion of PWH in phase 2 and phase 3 trials compared with early phase 1 and phase 1 trials is unclear but may be due to a delay in later phase trials adopting the FDA guidelines for including PWH in cancer clinical trials, a delay in regulators incorporating these guidelines, or both. Regarding funding, almost 75% of industry-sponsored and other-sponsored trials excluded PWH explicitly (74.3% and 74.1%, respectively), and while this was a higher proportion than National Insitutes of Health–sponsored trials (57.1%), the difference did not reach statistical significance.

Such disparities in access to clinical trials may be a result of existing limitations in where oncologic facilities are located. Onega et al^28^ previously showed that 13.9% of the general US population lived over 3 hours away from an NCI-designated facility in 2013. However, just 44.0% of HIV-inclusive trial sites and 58.2% of HIV-exclusive trial sites were located at an NCI-designated facility, with all other trial sites located at non-NCI designated facilities. A separate study by Onega et al^25^ in 2008 demonstrated that the median (IQR) travel time to oncologic care at any site (not just NCI-designated sites) in the US was just 13 (7-30) minutes, and while the South had slightly longer travel times than in the Northeast, the absolute difference was minimal (17 vs 9 minutes). Additionally, 94.8% of the population lived within 3 hours of any academic oncology center and 99.7% lived within 3 hours of any cancer facility.^25^ As such, the confluence of the HIV and regional disparities in CAR-T trial access are not inherent in cancer therapy but rather, we posit, reflect a lack of access to cutting-edge therapies in underresourced communities.

Historically, trials for CAR-T have excluded PWH. The trials used for approval of CAR-T as third-line therapy and second-line therapy for relapsed or refractory NHL all explicitly listed HIV seropositivity as a reason for exclusion.^2,3,4,5,6,7,8,9^ In our study, only 1 trial mentioned a justification for excluding PWH. This is despite recent FDA guidelines suggesting that persons with well-controlled HIV should not be excluded from cancer clinical trials on the basis of HIV alone.^29^ While there may be reasons to exclude PWH, such as an elevated viral load or opportunistic infections, these reasons should be explicitly stated, rather than a sweeping exclusion of PWH.

Notably, when assessing CD4^+^ cell counts, a surrogate marker often used to assess degree of immunodeficiency in PWH, in people without HIV undergoing CAR T-cell therapies, the median CD4^+^ count before CAR-T was 182 cells per mm^3^,^30^ not dissimilar to the pretreatment median CD4^+^ count of 228 cells per mm^3^ seen in a cohort study of PWH undergoing CAR-T cell therapies.^31^ Others have already demonstrated that PWH tolerate CAR-T safely.^31^

Overall, these results reflect access for CAR-T in the US for PWH. They may be transferrable to other marginalized populations in the US or in other countries; however, further studies are needed to evaluate this.

Despite recent FDA guidelines, many trials still exclude PWH from CAR-T clinical trials—just 1 of 58 trials excluding PWH specified a reason why in this study. Increasing access to cutting-edge clinical trials to address the growing cancer burden among PWH can be difficult, as smaller sites may lack adequate patient recruitment, infrastructure, funding, and staffing.^32^ Nevertheless, since longer travel times to CAR-T were previously shown to lead to less enrollment in trials and worse outcomes, it is clear that expanding access to clinical trials is of significant importance and could help ensure access to CAR-T for everyone, including PWH. Efforts to expand access to CAR-T trials may include decentralizing trials to sites in a wide geographical area or collaborations between community sites and academic sites. Certain clinical trials are already starting to explicitly include PWH^33^ or include cohorts of PWH^34^ and can serve as models for doing so. Focus should be placed especially in the South where disparities between PWH and persons without HIV are the greatest and the incidence of HIV is the highest.

### Limitations

This study has several limitations. First, it was assumed that all CAR-T clinical trials for which no mention of HIV was made included PWH since in practice we have seen that trials not mentioning HIV as an exclusion criterion often consider PWH with an undetectable viral load eligible. Second, there were no data available to estimate the percentage of patients that enrolled into an HIV-inclusive trial. It is very likely that some CAR-T clinical trials that were considered HIV-inclusive did not enroll any PWH. Together, these 2 factors mean access for PWH was likely overestimated. Sensitivity was performed to provide a lower bound on access for PWH, but future studies are needed to examine the true rates of enrollment of PWH in trials.

Additionally, population-weighted travel times used general population data including for PWH since zip code level data for PWH are not publicly available. This means travel time for PWH may be underestimated in areas of higher HIV prevalence. Other limitations of this model include not accounting for traffic time, not accounting for whether a patient relocates for a trial, and participants selecting a trial that is not the nearest available trial. Recent federal funding cuts may also have influenced which studies were actively recruiting at the time of study. Furthermore, most trials in this study included at least 2 types of lymphomas, which means access to each disease was not individually measured. Lymphomas that are not considered AIDS-defining may inherently have less emphasis on including PWH; however, even for lymphomas not associated with HIV, PWH should still be included at similar rates.

As a cross-sectional study, the study is limited in only capturing access at 1 time point. In addition, it is important to note that geospatial access is just one component of participants being able to take part in clinical trials, as there are multiple barriers to trial participation that were not analyzed in this study, including financial cost, lack of information about trial availability, and difficulty with systems navigation and logistics,^35^ all of which were beyond the scope of this study.

## Conclusions

In this cross-sectional study of travel time to trials for CAR-T for NHL, we found that PWH had to travel significantly longer to reach the nearest trial than the general population. Overall, these results highlight disparities in access to CAR-T by both geography and HIV status in the US for patients with NHLs, despite subtypes of NHL being consistently uniquely associated with HIV infection.

## Notes

Supplement 1. eFigure 1. Inclusion chart of all studies eligible for analysis. eFigure 2. Travel time to the nearest actively recruiting clinical trial by zip code. eTable 1. States without a CAR-T clinical trial. eTable 2. Median travel time to nearest trial for the general population by state. eTable 3. Median travel time to nearest trial that includes PWH by state. eTable 4. Median travel time to nearest trial that excludes PWH by state. eTable 5. Travel time to nearest clinical trial by inclusion or exclusion of PWH by income.

Supplement 2. Data Sharing Statement
