# Supplementary material for: Geospatial Access to CAR-T Clinical Trials for Non-Hodgkin Lymphoma for Persons With HIV
Source: JAMA Netw Open. 2026 May 22;9(5):e2614265. doi: 10.1001/jamanetworkopen.2026.14265 (PMC13197865; doi:10.1001/jamanetworkopen.2026.14265)
Supplement: Supplement 1. — eFigure 1. Inclusion chart of all studies eligible for analysis. eFigure 2. Travel time to the nearest actively recruiting clinical trial by zip code. eTable 1. States without a CAR-T clinical trial. eTable 2. Median travel time to nearest trial for the general population by state. eTable 3. Median travel time to nearest trial that includes PWH by state. eTable 4. Median travel time to nearest trial that excludes PWH by state. eTable 5. Travel time to nearest clinical trial by inclusion or exclusion of PWH by income. [file jamanetwopen-e2614265-s001.pdf]

Supplemental Online Content

Maillie L, Sisk M, Coghill AE, et al. Geospatial access to CAR-T clinical trials for non-Hodgkin lymphoma for persons with HIV. *JAMA Netw Open.* 2026;9(5):e2614265. doi:10.1001/jamanetworkopen.2026.14265

eFigure 1. Inclusion chart of all studies eligible for analysis.

eFigure 2. Travel time to the nearest actively recruiting clinical trial by zip code.

eTable 1. States without a CAR-T clinical trial.

eTable 2. Median travel time to nearest trial for the general population by state.

eTable 3. Median travel time to nearest trial that includes PWH by state.

eTable 4. Median travel time to nearest trial that excludes PWH by state.

eTable 5. Travel time to nearest clinical trial by inclusion or exclusion of PWH by income.

This supplemental material has been provided by the authors to give readers additional information about their work.

© 2026 Maillie L et al. *JAMA Network Open*.

eFigure 1. **Inclusion chart of all studies eligible for analysis**.

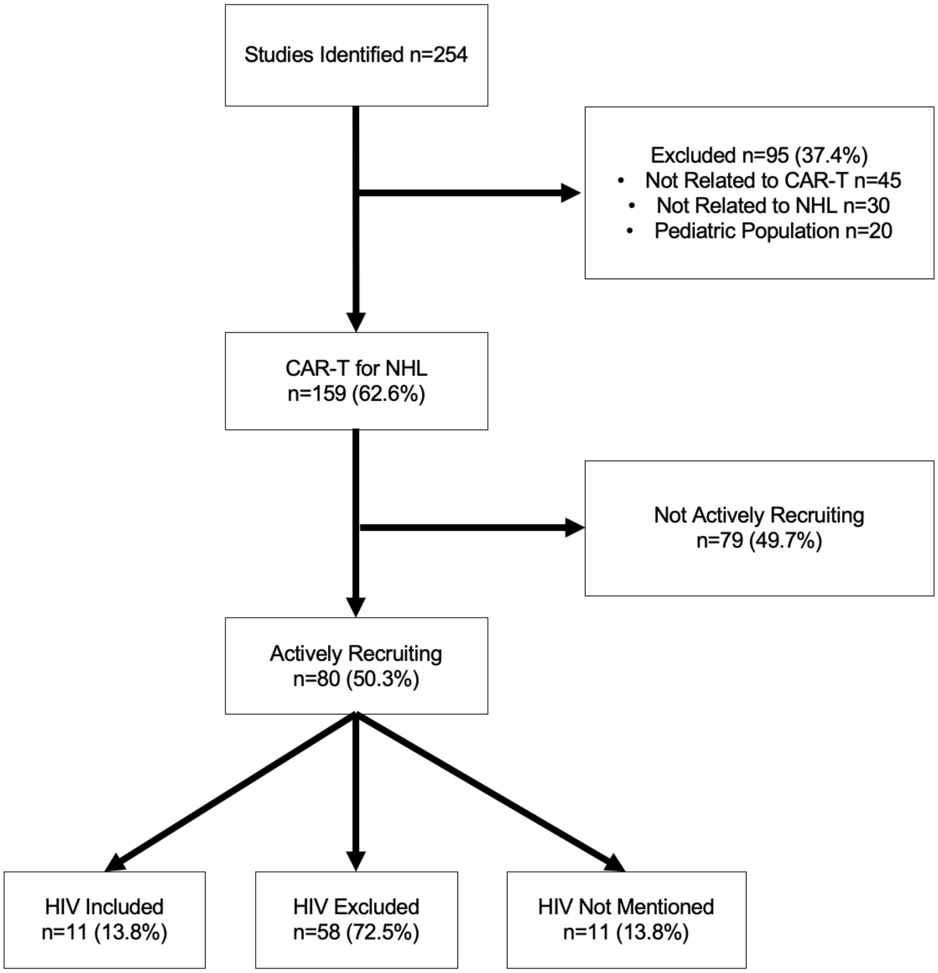

Percent is calculated using above level of inclusion as the denominator. CAR-T = Chimeric antigen receptor T-cell therapy; NHL = non-Hodgkin lymphoma; HIV = human immunodeficiency virus.

© 2026 Maillie L et al. *JAMA Network Open*.

eFigure 2. **Travel time to the nearest actively recruiting clinical trial by zip code.**

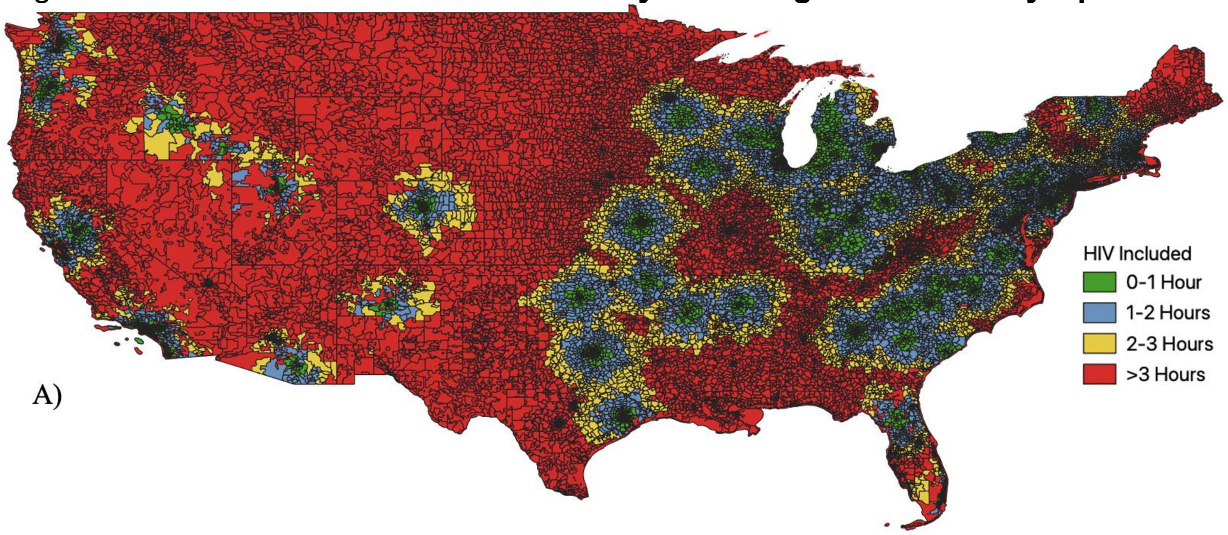

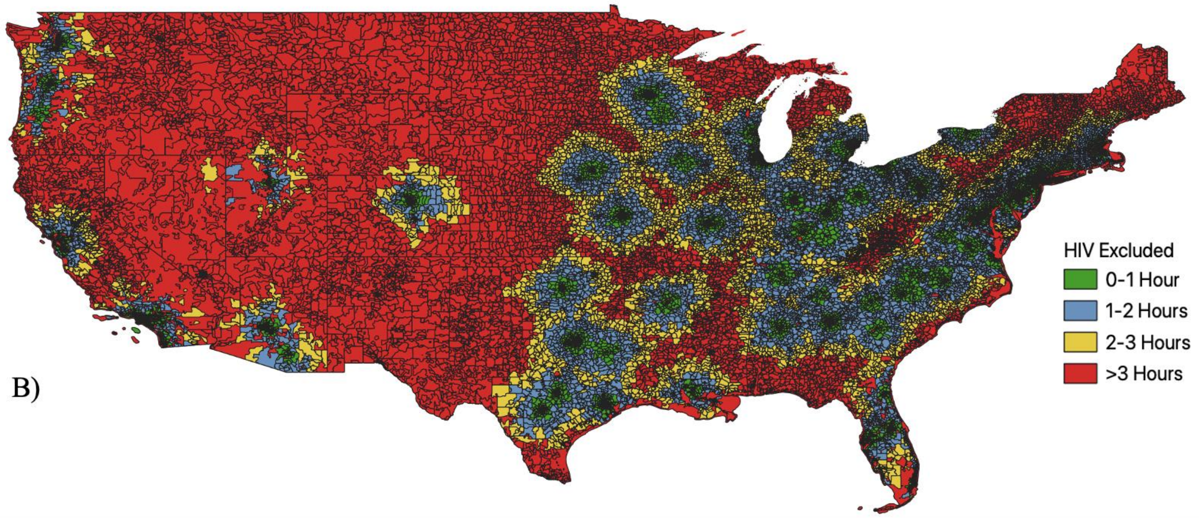

Results are shown for trials that included PWH (A) and trials that excluded PWH (B). HIV = human immunodeficiency virus; PWH = persons living with HIV.

© 2026 Maillie L et al. *JAMA Network Open*.

eTable 1. **States without a CAR-T clinical trial.**
Supplemental Tables:

**General Population**	**HIV-Inclusive**	**HIV-Exclusive**
Maine	Alabama	Idaho
Mississippi	Connecticut	Maine
Montana	Delaware	Mississippi
Nevada	District of Columbia	Montana
New Hampshire	Louisiana	Nevada
North Dakota	Maine	New Hampshire
Rhode Island	Mississippi	New Mexico
South Dakota	Missouri	North Dakota
West Virginia	Montana	Rhode Island
Wyoming	Nebraska	South Dakota
	Nevada	Vermont
	New Hampshire	West Virginia
	North Dakota	Wyoming
	Rhode Island	
	South Dakota	
	West Virginia	
	Wyoming	

Results are listed for the general population, trials that included PWH, and trials that excluded PWH.

© 2026 Maillie L et al. *JAMA Network Open*.

eTable 2. **Median travel time to nearest trial for the general population by state.**

State	Min	Q1	Med	Q3	Max	% 1-Hour	% 3-Hour
District of Columbia	0.00	0.15	0.21	0.25	0.33	100.0%	100.0%
New York	0.00	0.19	0.35	1.36	4.20	71.5%	97.2%
New Jersey	0.00	0.26	0.40	0.59	2.08	93.5%	100.0%
Delaware	0.00	0.26	0.45	1.38	2.32	68.0%	100.0%
Maryland	0.00	0.31	0.45	0.74	3.03	83.9%	100.0%
California	0.00	0.29	0.46	0.97	7.58	75.8%	93.4%
Arizona	0.00	0.32	0.50	0.94	8.54	77.3%	89.7%
Michigan	0.00	0.31	0.52	1.08	8.44	73.1%	96.4%
Oregon	0.00	0.26	0.54	1.73	6.64	64.9%	85.8%
Virginia	0.00	0.34	0.60	1.21	4.03	67.7%	97.5%
Minnesota	0.00	0.39	0.64	1.67	8.91	64.2%	87.1%
Illinois	0.00	0.30	0.64	1.33	3.50	66.7%	98.6%
Colorado	0.00	0.35	0.66	1.46	8.50	57.6%	88.5%
Connecticut	0.01	0.47	0.68	0.96	2.19	75.6%	100.0%
Texas	0.00	0.42	0.68	1.66	9.49	65.0%	85.1%
Massachusetts	0.00	0.29	0.69	1.07	3.13	70.0%	99.8%
Utah	0.00	0.44	0.71	1.22	9.37	66.0%	88.5%
Florida	0.00	0.43	0.78	1.39	5.69	61.3%	94.0%
Pennsylvania	0.00	0.37	0.85	1.40	3.43	56.5%	99.4%
Missouri	0.00	0.42	0.87	2.28	4.21	52.0%	94.8%
North Carolina	0.00	0.49	0.87	1.64	4.97	57.2%	95.2%
Kentucky	0.00	0.34	0.87	1.88	3.80	54.3%	98.2%
Ohio	0.00	0.40	0.87	1.31	3.07	56.7%	100.0%
South Carolina	0.00	0.42	0.88	1.68	5.31	52.2%	100.0%
Georgia	0.00	0.53	0.89	1.69	4.45	55.6%	95.8%
Washington	0.00	0.47	0.92	2.13	8.15	53.6%	80.2%
Indiana	0.00	0.48	0.94	1.51	2.64	53.4%	100.0%
Wisconsin	0.00	0.41	0.97	1.99	5.49	50.7%	92.8%
Tennessee	0.00	0.47	0.98	1.87	3.49	50.6%	98.4%
Nebraska	0.06	0.34	1.09	2.35	7.06	46.8%	83.1%
Arkansas	0.00	0.48	1.13	1.77	3.49	45.0%	97.8%
Rhode Island	0.94	1.07	1.20	1.37	2.85	12.7%	100.0%
Vermont	0.00	0.50	1.21	2.16	3.66	43.8%	94.7%
Idaho	0.00	0.30	1.26	3.72	10.29	48.5%	61.1%
New Hampshire	0.79	1.11	1.39	1.94	4.07	13.0%	97.4%
New Mexico	0.00	0.40	1.48	3.99	6.85	42.4%	61.1%
Oklahoma	0.00	0.46	1.64	1.97	6.32	36.7%	97.0%
Louisiana	0.00	1.27	1.64	2.23	4.05	19.0%	89.4%
Alabama	0.00	1.19	1.88	2.55	4.91	21.9%	77.8%

© 2026 Maillie L et al. *JAMA Network Open*.

Iowa	0.00	1.21	1.97	2.32	4.04	19.3%	93.6%
**State**	**Min**	**Q1**	**Med**	**Q3**	**Max**	**% 1-Hour**	**% 3-Hour**
Kansas	0.00	0.45	2.08	2.98	6.39	35.4%	75.3%
West Virginia	0.95	1.90	2.62	3.17	4.42	1.6%	66.9%
Mississippi	0.54	2.14	2.87	3.37	4.81	7.2%	58.2%
Maine	1.27	2.27	3.15	4.38	9.41	0.6%	46.8%
Nevada	0.95	4.02	4.29	4.43	8.10	0.0%	13.9%
South Dakota	2.00	3.35	4.63	7.38	10.76	0.0%	3.9%
Wyoming	1.91	2.90	4.91	6.45	9.43	0.1%	25.4%
North Dakota	3.89	4.76	7.66	9.36	12.34	0.0%	0.1%
Montana	4.90	7.51	8.90	9.57	16.50	0.0%	0.0%

States are sorted from shortest to longest median travel time.

© 2026 Maillie L et al. *JAMA Network Open*.

eTable 3. **Median travel time to nearest trial that includes PWH by state.**

State	Min	Q1	Med	Q3	Max	% 1-Hour	% 3-Hour
New Jersey	0.00	0.37	0.52	0.74	2.08	88.5%	100.0%
New York	0.00	0.26	0.55	1.50	4.20	64.9%	94.9%
Michigan	0.00	0.32	0.57	1.12	8.44	70.7%	96.4%
California	0.00	0.35	0.60	1.20	7.75	67.6%	93.3%
Illinois	0.00	0.31	0.69	1.96	5.71	62.2%	85.3%
Colorado	0.00	0.40	0.70	1.48	8.50	57.2%	88.4%
Massachusetts	0.00	0.35	0.71	1.14	3.37	67.1%	99.3%
Utah	0.00	0.44	0.71	1.22	9.37	66.0%	88.5%
Maryland	0.05	0.49	0.81	1.15	3.49	64.7%	98.7%
Oregon	0.00	0.30	0.83	2.44	7.30	53.8%	77.7%
South Carolina	0.00	0.42	0.88	1.70	5.31	52.2%	100.0%
Kentucky	0.00	0.37	0.89	2.30	4.11	53.8%	90.6%
North Carolina	0.00	0.51	0.89	1.73	6.63	55.2%	93.9%
Pennsylvania	0.00	0.46	0.92	1.46	3.44	53.4%	99.3%
Georgia	0.00	0.53	0.92	1.85	4.45	53.9%	93.4%
Ohio	0.00	0.44	0.92	1.36	3.07	54.6%	100.0%
Washington	0.00	0.47	0.92	2.13	8.15	53.6%	80.2%
Indiana	0.00	0.56	0.95	1.60	2.64	52.6%	100.0%
District of Columbia	0.85	0.93	0.97	1.01	1.14	69.2%	100.0%
Wisconsin	0.00	0.41	1.03	2.17	6.74	49.2%	90.8%
Arkansas	0.00	0.48	1.13	1.77	3.58	45.0%	97.6%
Delaware	0.59	0.96	1.14	2.02	3.08	34.9%	99.9%
Vermont	0.00	0.50	1.21	2.16	3.74	43.8%	94.2%
Idaho	0.00	0.30	1.26	3.72	10.29	48.5%	61.1%
Rhode Island	1.02	1.15	1.28	1.45	2.93	2.5%	100.0%
Texas	0.00	0.57	1.33	3.55	10.84	45.4%	64.6%
New Hampshire	0.79	1.11	1.40	1.95	4.07	13.0%	97.4%
New Mexico	0.00	0.40	1.48	3.99	6.85	42.4%	61.1%
Virginia	0.03	1.24	1.52	2.02	4.38	15.9%	96.7%
Oklahoma	0.00	0.46	1.64	1.97	6.42	36.7%	96.7%
Connecticut	0.52	1.34	1.80	2.10	3.46	10.3%	100.0%
Minnesota	0.00	1.62	1.88	2.87	10.45	5.4%	76.5%
Kansas	0.00	0.45	2.08	3.00	6.66	35.4%	75.0%
Iowa	0.00	1.46	2.13	2.66	5.38	15.6%	79.4%
Arizona	0.00	1.91	2.22	2.58	8.54	14.9%	84.4%

© 2026 Maillie L et al. *JAMA Network Open*.

Florida	0.00	1.02	2.23	2.80	6.69	24.8%	82.6%
**State**	**Min**	**Q1**	**Med**	**Q3**	**Max**	**% 1-Hour**	**% 3-Hour**
West Virginia	1.00	2.03	2.74	3.28	4.63	1.2%	62.4%
Missouri	0.06	1.40	3.12	4.53	5.83	19.9%	47.7%
Maine	1.27	2.27	3.15	4.38	9.41	0.6%	46.8%
Tennessee	0.00	2.41	3.19	3.55	4.50	14.6%	41.4%
Alabama	1.47	3.03	3.57	4.23	7.09	0.0%	22.3%
Nebraska	2.20	3.64	3.80	4.95	8.35	0.0%	1.4%
Mississippi	0.54	2.59	4.22	5.87	7.72	7.2%	29.7%
Nevada	0.95	4.28	4.62	4.75	8.10	0.0%	13.9%
Wyoming	1.91	2.90	4.91	6.45	9.43	0.1%	25.4%
Louisiana	2.38	4.06	5.34	6.47	8.49	0.0%	3.7%
South Dakota	4.21	4.61	5.91	7.46	12.00	0.0%	0.2%
Montana	4.90	7.51	8.90	9.57	16.50	0.0%	0.0%
North Dakota	5.45	6.32	9.22	10.91	13.90	0.0%	0.1%

States are sorted from shortest to longest median travel time.

© 2026 Maillie L et al. *JAMA Network Open*.

eFigure 4. **Median travel time to nearest trial that excludes PWH by state**.

**State**	**Min**	**Q1**	**Med**	**Q3**	**Max**	**% 1-Hour**	**% 3-Hour**
District of Columbia	0.00	0.15	0.21	0.25	0.33	100.0%	100.0%
New York	0.00	0.19	0.35	1.40	6.43	71.4%	93.8%
New Jersey	0.00	0.26	0.40	0.59	2.08	93.5%	100.0%
Delaware	0.00	0.26	0.45	1.38	2.32	68.0%	100.0%
Maryland	0.00	0.31	0.46	0.77	3.03	83.9%	100.0%
Arizona	0.00	0.32	0.50	0.95	8.61	77.3%	89.7%
California	0.00	0.31	0.51	1.42	7.58	68.6%	92.2%
Virginia	0.00	0.34	0.61	1.21	4.03	67.7%	97.5%
Minnesota	0.00	0.39	0.64	1.67	8.91	64.2%	87.1%
Colorado	0.00	0.35	0.66	1.46	8.75	57.6%	88.5%
Connecticut	0.01	0.47	0.68	0.96	2.19	75.6%	100.0%
Texas	0.00	0.42	0.68	1.66	9.49	65.0%	85.1%
Massachusetts	0.00	0.29	0.69	1.07	3.13	70.0%	99.8%
Oregon	0.00	0.44	0.76	1.97	8.69	56.6%	83.7%
Illinois	0.00	0.39	0.79	1.44	3.50	60.7%	98.2%
Florida	0.00	0.44	0.81	1.52	6.05	58.5%	92.8%
Utah	0.00	0.49	0.83	1.33	9.44	63.2%	88.4%
Ohio	0.00	0.42	0.87	1.31	3.07	56.1%	100.0%
Kentucky	0.00	0.36	0.88	1.89	3.80	54.2%	97.9%
North Carolina	0.00	0.49	0.88	1.73	4.97	55.7%	95.0%
Georgia	0.00	0.53	0.90	1.71	4.77	55.2%	94.4%
Washington	0.00	0.49	0.93	2.13	8.15	53.6%	80.2%
Missouri	0.00	0.46	0.94	2.60	4.73	51.0%	82.7%
Pennsylvania	0.00	0.41	1.04	1.79	4.17	48.4%	95.4%
Nebraska	0.06	0.34	1.09	2.35	7.06	46.8%	83.1%
Michigan	0.00	0.50	1.10	2.35	8.44	47.8%	87.5%
Indiana	0.00	0.53	1.12	1.84	3.00	44.5%	100.0%
Rhode Island	0.94	1.07	1.20	1.37	2.85	12.7%	100.0%
New Hampshire	0.79	1.11	1.39	1.99	5.13	13.0%	95.2%
Wisconsin	0.00	0.70	1.51	2.20	5.85	34.7%	88.7%
South Carolina	0.00	0.77	1.52	2.99	6.23	33.8%	75.4%
Louisiana	0.00	1.27	1.64	2.23	4.05	19.0%	89.4%
Tennessee	0.00	0.73	1.68	2.72	3.76	36.1%	86.8%
Oklahoma	0.00	0.46	1.77	2.20	6.36	36.3%	91.5%
Alabama	0.00	1.19	1.88	2.55	4.91	21.9%	77.8%

© 2026 Maillie L et al. *JAMA Network Open*.

Iowa	0.00	1.21	1.97	2.33	4.04	19.3%	93.6%
**State**	**Min**	**Q1**	**Med**	**Q3**	**Max**	**% 1-Hour**	**% 3-Hour**
Kansas	0.00	0.56	2.24	3.01	6.39	33.9%	73.1%
Arkansas	0.00	0.92	2.25	3.11	4.18	26.5%	73.2%
West Virginia	0.95	1.90	2.62	3.19	4.44	1.6%	66.3%
Maine	1.27	2.27	3.15	4.38	9.74	0.6%	46.8%
Mississippi	1.32	2.82	3.20	3.55	4.81	0.1%	38.6%
Vermont	2.37	3.52	4.08	4.35	5.36	0.0%	12.4%
Nevada	2.97	4.22	4.36	4.49	8.96	0.0%	0.1%
South Dakota	2.00	3.35	4.63	7.38	10.76	0.0%	3.9%
Wyoming	1.91	2.90	4.91	6.46	9.43	0.1%	25.4%
Idaho	2.35	4.33	5.84	6.20	11.76	0.0%	1.4%
New Mexico	3.07	6.84	7.62	7.84	9.45	0.0%	0.2%
North Dakota	3.89	4.76	7.66	9.36	12.34	0.0%	0.1%
Montana	5.46	8.33	9.46	10.00	16.50	0.0%	0.0%

States are sorted from shortest to longest median travel time.

© 2026 Maillie L et al. *JAMA Network Open*.

			Median Population-Weighted Travel Time (IQR) in Hours		One-Hour Access (%)			Three-Hour Access (%)		
**Region**		**Population in Millions (%)**	**HIV Included**	**HIV Excluded**	**HIV Included**	**HIV Excluded**	**p-value**	**HIV Included**	**HIV Excluded**	**p-value**
**Northeast**										
	Q1 (Lowest)	7.6 (16.7)	0.73 (0.24-1.78)	0.65 (0.19-1.83)	53.0	56.2	<0.001	93.4	90.0	<0.001
	Q2	9.7 (21.3)	1.04 (0.38-1.82)	0.99 (0.30-2.02)	49.5	50.3	<0.001	94.6	88.7	<0.001
	Q3	11.9 (26.0)	0.99 (0.41-1.61)	0.79 (0.35-1.50)	50.0	56.1	<0.001	97.2	95.7	<0.001
	Q4 (Highest)	16.4 (36.0)	0.65 (0.39-1.05)	0.42 (0.25-0.76)	72.9	84.1	<0.001	99.1	98.9	<0.001
**Midwest**										
	Q1 (Lowest)	13.7 (25.7)	1.36 (0.35-2.51)	1.34 (0.33-2.33)	38.8	39.0	<0.001	81.1	87.0	<0.001
	Q2	15.8 (29.7)	1.45 (0.72-2.82)	1.45 (0.68-2.42)	33.9	32.5	<0.001	77.2	85.8	<0.001
	Q3	13.9 (26.0)	0.98 (0.51-2.09)	0.86 (0.48-1.73)	50.7	57.1	<0.001	83.8	92.6	<0.001
	Q4 (Highest)	9.9 (18.6)	0.72 (0.43-1.71)	0.63 (0.45-0.94)	65.1	76.7	<0.001	89.7	97.8	<0.001
**South**										
	Q1 (Lowest)	33.3 (34.3)	2.15 (1.01-3.27)	1.67 (0.70-2.66)	24.7	32.3	<0.001	69.1	82.4	<0.001
	Q2	25.0 (25.7)	1.94 (0.80-3.05)	1.09 (0.53-2.00)	31.1	47.1	<0.001	73.6	88.8	<0.001
	Q3	20.9 (21.5)	1.25 (0.57-2.76)	0.71 (0.41-1.42)	44.7	65.9	<0.001	77.6	91.5	<0.001
	Q4 (Highest)	18.0 (18.5)	0.94 (0.59-1.71)	0.57 (0.40-0.80)	53.5	83.8	<0.001	85.7	96.0	<0.001
**West**										
	Q1 (Lowest)	9.1 (15.5)	2.19 (0.47-3.78)	2.09 (0.39-4.27)	30.2	35.1	<0.001	65.1	59.1	<0.001
	Q2	13.2 (22.5)	1.36 (0.45-3.17)	1.40 (0.40-3.73)	43.8	45.0	<0.001	73.2	68.2	<0.001
	Q3	17.1 (29.0)	0.82 (0.38-1.94)	0.73 (0.37-1.90)	55.2	56.6	<0.001	85.1	81.6	<0.001
	Q4 (Highest)	19.4 (33.0)	0.60 (0.35-1.06)	0.50 (0.33-0.84)	72.2	80.4	<0.001	96.5	95.5	<0.001

eTable 5. **Travel time to nearest clinical trial by inclusion or exclusion of PWH by income.**

Comparison of the median population-weighted travel time to nearest CAR-T clinical between trials enrolling PWH and trials that excluded PWH, as well as one-hour and three-hour access metrics, by U.S. region and median household income for each zip code. Q1 <$50,828, Q2 = $50,828-$64,678, Q3 = $64,679-$85,188, and Q4 >$85,189. Q = quartile; IQR = interquartile range; HIV = human immunodeficiency virus.

© 2026 Maillie L et al. *JAMA Network Open*.
