# Supplementary material for: Geospatial Access to CAR-T Clinical Trials for Non-Hodgkin Lymphoma for Persons With HIV
Source: JAMA Netw Open. 2026 May 22;9(5):e2614265. doi: 10.1001/jamanetworkopen.2026.14265 (PMC13197865; doi:10.1001/jamanetworkopen.2026.14265)
Supplement: Supplement 2. — Data Sharing Statement [file jamanetwopen-e2614265-s002.pdf]

Data Sharing Statement

Maillie. Geospatial Access to CAR-T Clinical Trials for Non-Hodgkin Lymphoma for Persons With HIV. *JAMA Netw Open*. Published May 22, 2026.
doi:10.1001/jamanetworkopen.2026.14265

Data
Data available: Yes
Data types: Data (not involving human participants)
How to access data: All clinical trial data were extracted from clinicaltrials.gov, and all roadway network data including road speeds were taken from OSRM (https://project-osrm.org/). Population data from the 2020 U.S. Census and the 2016-2020 American Community Survey are available online (www.census.gov). A complete list of clinical trials used for analysis as well as the transportation network used for analysis will be made available upon request by emailing the corresponding author at lukemaillie18@gmail.com.
When available: With publication

Supporting Documents
Document types: None

Additional Information
Who can access the data: Anyone requesting data
Types of analyses: For any purpose
Mechanisms of data availability: With a signed data access agreement
